# Cultural participation and life satisfaction: an investigation across different family backgrounds

**DOI:** 10.1371/journal.pone.0346887

**Published:** 2026-04-10

**Authors:** Romain Lerouge, Michela Arnaboldi

**Affiliations:** Management Engineering, Politecnico di Milano, Milano, Italy; University of Naples Federico II: Universita degli Studi di Napoli Federico II, ITALY

## Abstract

The economic and social impact of cultural participation has recently become once more a subject of interest for policy-making, especially as a driver potentially affecting social inclusion and quality of life. Two topics of interest emerge in research on this subject. The first topic concerns the relationship between cultural experiences and life satisfaction, this being a comprehensive measure of the highest human motivation. The second topic relates to the multi-faceted relationship between cultural participation and family background. According to previous studies, these two topics are strictly intertwined; however, they have not been analysed in an integrated manner. Drawing on equality of opportunity theory, this study integrates both perspectives to provide a general overview on the link between cultural participation and life satisfaction for on people coming from different family backgrounds. Research based on a survey disseminated in Italy (N = 1 235) was conducted to investigate if and how family background moderates people’s ability to achieve life satisfaction through their involvement in cultural events. The results support customised policies that introduce a new approach to assessing the impact of cultural participation, thereby disclosing the role it plays in disadvantaged backgrounds and highlighting the cultural events responsible for the strongest benefits.

## Introduction

In recent years, cultural participation is re-emerging as a crucial subject of interest for the socio-economic impact it could generate [1–3]. The Organisation for Economic Co-operation and Development (OECD), the European Commission and a larger number of government and public institutions are increasingly basing their policies in this area on the promotion of cultural participation, calling for further research on the topic [4–6]. The development of more tailored policies is believed to engender significant benefits not only in terms of people’s intellectual enrichment, but also in terms of social inclusion [7]. This is indeed a crucial issue considering that cultural participation has been depicted in the past as selective and restricted to advantaged social classes [8,9].

Two main areas of research that explore the socio-economic benefits of cultural participation can be identified. First, a growing body of research portrays cultural participation as a means to improve quality of life [10–12]. Aside from the long-standing approach linking cultural participation to economic resources, usually expressed through GDP per capita [13], several recent studies cover a wide range of positive externalities that take in individual values such as civic engagement [14], personal health [11] and social cohesion [15]. Under this approach, a recent stream of studies investigates the relationship between cultural participation and people’s well-being, especially focusing on its long-term cognitive component, which is life satisfaction [3,10,16]. Life satisfaction is intended as a person’s self-judgment on their life which reflects their values and goals, and it is believed to be the highest level of human motivation [17] and a good proxy of individual utility [16].

The association between cultural participation and life satisfaction has been investigated in previous studies mainly focusing on a large spectrum of passive cultural activities, which take the form of attendance to events or experiences such as theater shows or museum visits [11,18]. While some studies have observed a positive link with life satisfaction [10,16,19], the relationship has been described in other studies as weak [20] or even non-significant [21]. Overall, our understanding of the dynamics of this association is still considered preliminary, insufficient and poorly structured [12], with few investigations on which of cultural events categories arouse the strongest benefit [3,10,16].

On the other side, people’s cultural participation also emerges as a factor strictly tied to social inequalities, a major concern in public and political debate [1,22–24]. Promoting equal opportunities is considered a crucial priority for public agents [25], also considering citizens’ increasing sensitiveness towards inequalities [26]. The existence of potential disparities is well conceptualised and framed in Equality of Opportunity theory [27], aiming at measuring the extent to which people’s outcomes are affected by exogenous and circumstantial factors lying beyond their specific responsibility, mainly related to family background [28]. This phenomenon is typically investigated looking at the influence of family background on people’s income [25,29,30] and, more recently, on well-being [30,31], due to the higher comprehensiveness of this metric.

Despite policy depicting cultural participation as an instrument to promote social inclusion [7], research highlights how the benefits from consuming and interacting with culture are thus mainly absorbed by the most advantaged groups in the population [7,22]. Recent studies observed indeed the key role of parents in transmitting cultural participation habits [32,33], thereby selectively benefiting a few citizens, whilst widening the disparity gap [9]. However, in existing literature investigating the potential benefits of cultural participation in life satisfaction, family history often plays a marginal role, implicitly assuming that people are equally able to access and benefit from cultural activities, and neglecting the existing differences in life satisfaction between advantaged and disadvantaged family backgrounds [30,34].

The existing literature overlooks two points covered in this section. The first point is that there is no perspective on the vast and diverse range of cultural activities that people can access and how these different events variously relate to life satisfaction. The second neglected point is that, when assessing the potential effect of cultural participation, family background does not enter the analysis as a significant moderating factor. Starting from these considerations, the objective of this study is to investigate the relationship between cultural participation and life satisfaction, assessing whether it is shaped by family background.

The research questions are thus defined as follows

RQ.1 To which extend does the participation to different typologies of cultural activities is related to life satisfaction?
RQ.2 What is the role of family background in moderating the relationship between cultural participation and life satisfaction?

In order to address this objective, a survey was designed and disseminated receiving 1 235 replies from a statistically representative sample of the Italian adult population with access to the internet in terms of age, gender and geographical area. Italy is chosen as the empirical setting because of its rich landscape of cultural facilities and events, coupled with the existence of rooted inequalities of opportunity [25,35]. Through the means of factor analysis, two sub-groups of similar cultural activities were first identified, and Propensity Score Matching (PSM) was then used to explore how each of them relates to life satisfaction. Specifically, this model was extended to take in the simultaneous influence exerted by both cultural participation and family background on life satisfaction outcomes. This novel methodological approach is proposed to limit confounding biases for both the impact and the moderating factor, describing at the same time the inequalities in life satisfaction and the different links between cultural participation and life satisfaction for people from advantaged and disadvantaged family backgrounds.

This paper is structured as follows. Firstly, the literature review delves into the main subject matter covered in the study and associated research questions. The methodology section sets out the data collected, the steps in the analysis and the models employed, including a detailed overview of the procedures applied to extend PSM model. This part leads to the description of the main results, which are discussed in light of the objectives of the study. The article concludes with considerations on the implications for policy making.

## Literature review

The subject of this research is cultural participation, which includes all the different activities relating to the production and/or reception of human expressions that emerge from the domain of human imagination [36]. Cultural events satisfy the human thirst for knowledge, aesthetics and self-realisation [20] and can be connected to the main domains of cultural heritage, performing arts, visual arts, books and literature, and audio-visual media [37]. Within the broad and blurred scope of cultural participation [21], the term receptive activity is used for a cultural event or experience that implies attendance, such as going to a concert or a museum [38].

Cultural participation, and in particular receptive activities, has been studied extensively for its role in affecting and reinforcing social positions. While cultural consumption is seen as a signal for more developed creativity [39], a stream of studies that refer back to [9] seminal work is concerned with the significant influence exerted by a person’s family background. Cultural participation is described as a means of social distinction, where high-end culture is mainly absorbed by a few social layers, encouraging the selective distribution of social capital and accentuating inequality [7,22]. The connection between cultural participation and social belonging, mainly intended in terms of family background, has been explored in several studies [32,39]. Previous findings show some association between attending cultural events and social capital, despite the criticism that cultural participation has lost its standing as a means of social distinction in the post-industrial world, with the ensuing increase in general prosperity [8].

Aside from this first debate, a growing community of practitioners has been addressing the overall shift in values towards more rounded ideals, where people play a relevant role in determining their own self-expression, lifestyle and objectives [8] and well-being is becoming a central priority for many governments and institutions [2,40,41]. An increasing body of academic research is concerned with analysing the effect of cultural participation on life satisfaction [10,16,42], representative of people’s values and of individual utility [16,43]. Life satisfaction is indeed believed to be the strongest incentive for human action and motivation [17]. Referring to self-determination theory, the level of motivation is affected by three fundamental needs, which are a sense of competence, perceived autonomy and relationships with others [44], and all three could, in turn, be affected by cultural participation.

Studies that look into the positive correlation between cultural participation and life satisfaction have considered a wide array of events belonging to the cultural sphere. These works highlight a relationship that varies according to factors such as frequency of participation, where regularity seems to be relevant [45], gender, where women seem to gain greater benefit [20], and there is even a place for religious faith [46]. Aside from these results, numerous studies refer to the many limitations in existing knowledge on the subject. The topic is still seen as understudied [11,12,45], badly structured and formalised [20,46], and undervaluing the potential role of other factors potentially affecting this relationship [10,11,47,48]. In this heterogeneous stream of studies, one significant factor only marginally considered in the Bourdieusian perspective is the influence of family background, despite it playing a significant role in shaping participants’ cultural habits and social capital [9]. Additionally, it is not clear if the link varies by type of cultural activity, and thus whether some events are more likely to be more significantly related than others [3,10,12]. The extant studies either focus singularly on a few typologies of events [16,20,47] or propose aggregated measures that bundle a wide array of different ones together [21,38,49].

**Fig 1:**
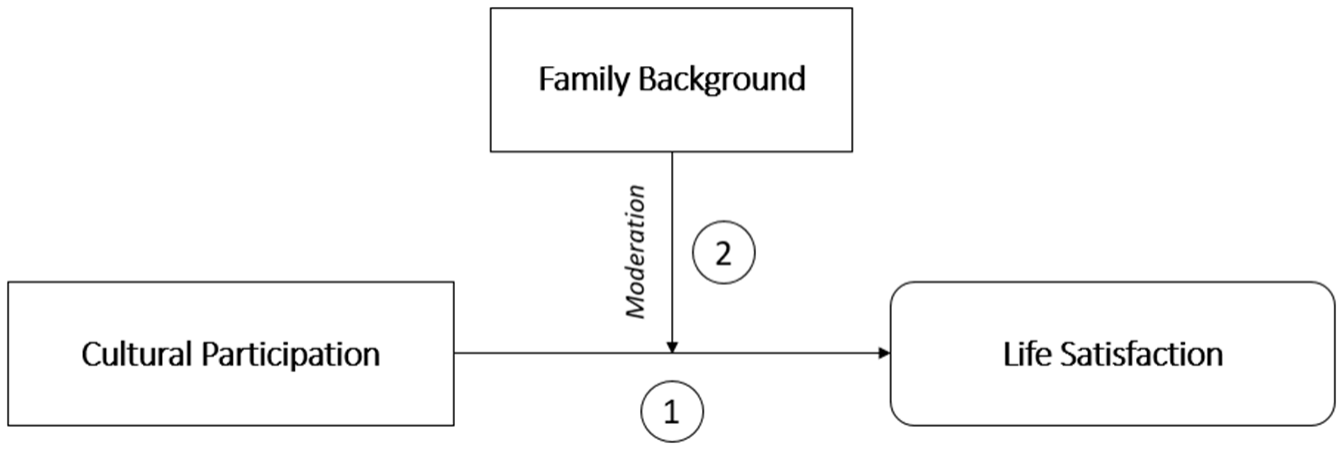
Theoretical framework.

Starting from these considerations, this study investigates the relationship between cultural participation and life satisfaction, in order to assess whether family background shapes a person’s ability to benefit from their cultural involvement. The underlying assumption of this study is that focusing solely on cultural participation is not enough to explain its relationship with life satisfaction, since different family backgrounds could spawn different occasions and opportunities. This investigation sheds light on the role of cultural participation in the characterisation of inequalities of opportunity [27] in life satisfaction. The authors posit that to identify the true effect brought by cultural participation, it is important to also account for the portion of life satisfaction that is already explained by family background, which is deemed as the most precise proxy to express the influence of exogenous factors that lie behind individual responsibility [25,28]. Uncovering the various opportunities available for people from different backgrounds could bring insights useful for policy-makers who are more interested in addressing structural inequalities than in the overall difference in outcomes achieved [25]. The theoretical framework of the study is shown in Figure 1. This setup with the moderating role of family background is becoming increasingly used by studies in various domains [50–52].

## Materials and methods

The objective of the analysis is to study the relationship between cultural participation and life satisfaction, assessing whether it is shaped by family background. Methodologically, the aim is to test the moderating effect exerted by family background, defined as the effect played by a moderator variable that affects the strength and/or direction of the relationship between a predictor variable (i.e., cultural participation) and an outcome variable (i.e., life satisfaction) [53].

The analysis is structured into two parts, following the two steps that are required to test a moderation effect, and reflecting our two research questions. In the first part, the study tests the relationship between cultural participation and life satisfaction. To account for the heterogeneity of cultural events, this study identifies sub-categories of similar events and investigates their respective link with life satisfaction through PSM. The second part of the analysis explores the mixed effect of cultural participation and social background on life satisfaction, determining the size of the moderation. The findings are expected to show how family background fashions a person’s ability to benefit from the same types of cultural participation previously identified.

### Survey design

To conduct the research, a survey was sent out to a sample of people across Italy between the 28th of April and the 6th of May 2022. Italy was chosen because of its rich and diverse cultural landscape of facilities and events, as well as being beset with nation-wide social imbalance and inequality of opportunity [25]. The survey was designed by the authors together with the support of Digital Innovation for Culture Observatory of Politecnico di Milano with the goal of mapping people’s cultural habits. It was then disseminated by Doxa (https://www.bva-doxa.com/), an external provider specialized in customer surveys and market research, using Computer Assisted Web Interviewing (CAWI). Answers are completely anonymous and do not require any form of consent. Data was collected and provided to the authors in an anonymous form. The target is set as the adult national population with access to the internet (accounting for approximately 91.4% of the Italian population in 2022 [54]). This latter specification is due to the nature of the CAWI methodology of the survey, which was disseminated online and thus targeted the population endowed with some access to the internet in Italy. Moreover, only adults were included, thereby assuming that they have more freedom to decide their level of engagement in the arts and to select the cultural activities they may prefer.

The final cleaned sample contains 1 235 anonymous questionnaires, and the relative respondents are statistically representative of the Italian population with access to the internet, in terms of age (considering people between 18 and 75 years of age), gender and geographical area. The variables included in the questionnaire are described in the next sections.

Considering the theoretical framework of the study, the analysis is based on the three variables of cultural participation, life satisfaction and family background.

To measure cultural participation, this study covers a large spectrum of receptive (also called “passive” or “audience-based”) activities. These events can be addressed directly by the public policy-makers responsible for cultural infrastructures and also a person’s decision to take part in most cultural events is expected to be significantly influenced by their family background [9,49]. The respondents’ receptive cultural participation was measured for 11 events, taking as reference the set of events identified in the extant literature, which include both classic and modern events [55]. The receptive activities selected are museums visits, classical concerts and opera, theatre shows, ballet and traditional dance performances, music and dance festivals, cabaret and stand-up comedy, cinema, pop and rock concerts, library and archives, clubbing, circus and open-air cultural events (e.g., historical re-enactments). The respondents were asked to say whether they had attended each type of event in the past twelve months and frequency was measured on a four-point scale, “never”, “once in the past year”, “a few times in the past year”, “a few times a month”.

Life satisfaction has been measured in many studies, and these have developed and proposed several evaluation scales [43,56–58]. In general, well-being can be seen as the combination of two principal elements. One is what is known as the pleasant and unpleasant effect, which relates to the respondent’s mood at that moment, and the other is life satisfaction, which is instead the respondent’s appraisal of their overall quality of life. Many studies, including this one, concentrate only on the second element, life satisfaction, which is believed to be more stable in time, being the effect of cognitive and conscious motives, and so more comprehensive of a person’s general situation and less subject to transitory moods [17,59]. In this study, the focus on life satisfaction is measured through the validated and widely used Satisfaction With Life Scale (SWLS) [17]. This scale consists of five questions to be answered on a 7-point Likert scale. The level of self-assessed life satisfaction is thus the sum of the associated five scores.

Family background is a crucial variable that could moderate the effect of cultural participation and play a role in characterising equalities of opportunity. Referring to the methodology of the large existing literature on the subject [25,29,60] and on sociological studies on inequalities in arts consumption [39], the family background is estimated with parental education, defined as the highest level of education attained by the parents of the respondent. Although family background is characterised by several diverse factors (e.g., ethnicity or nationality), this metric is capable of grasping and synthesising the greatest part of its variability [28], becoming a very recurrent metric in existing studies mapping inequalities of opportunity [29,32]. Consequently, one question in the survey asks the respondents for information about their parents’ level of education (specifically, the parent with the highest qualifications), on a 4-item scale from “no formal education or primary education” to “master or postgraduate degree”.

The data include a set of confounding variables to map some of the respondents’ demographic, personal and behavioural features, and so limit a potential omitted variable bias. The survey questions covered the respondents’ age, gender, education, geographical area of residence, population of the city of residence and type of job/employment. This latter variables consist of 21 distinct typologies that are collapsed in regression models into four categories: “worker,” “unemployed,” “student” and “retired.” The respondents’ income was measured on a 10-band income scale, ideally following the deciles of income ranges in Italy [61]. The respondents were asked to give their monthly income net of tax, as it provides more precise information [62]. The 10 levels go from “I have no monthly income” to “more than 10,000 euros per month”. Given the sensitivity of this matter, the respondents could also choose not to answer. Two further measures were added: religious faith, given its potential associations with the main variables in the theoretical framework [46] and household size.

The variables in the survey are all described briefly in Table 1, apart from job/occupation and participation rates for each of the 11 events considered, both of which are in the Supporting information Section (see S1 Table and S2 Table).

**Table 1:** Descriptive sample statistics.

Variables	N	Mean	St. Dev.	Min	Max
Age	1 235	44.530	13.778	18	75
Life Satisfaction	1 235	23.219	6.089	5	35
Income range [1 – 10]	1 007	4.596	1.652	1	10
Education level	1 235	4.560	1.096	1	7
Household size	1 235	2.990	1.205	1	4
City population	1 235	3.753	1.559	1	6
	N	%			
Gender	1 235				
*Male*		50.1			
*Female*		49.9			
Region	1 235				
*North-West*		26.8			
*North-East*		19.8			
*Centre*		20.0			
*South*		23.0			
*Islands*		10.4			
Parents highest level of education	1 235				
*Primary school or below*		12.5			
*Lower secondary/Middle school*		25.4			
*Higher secondary/High school*		39.5			
*University or above*		22.6			
Religious faith	1 235				
*Believer*		59.1			
*Atheist*		20.5			
*Agnostic*		13.3			
*Don’t know*		7.1			
Occupation	1 235				*See supp. information*
Participation in cultural events	1 235				*See supp. information*

### Empirical models

The study is structured into two main parts. The first focuses on the average link between cultural participation (CP) categories and life satisfaction, and the second looks at the moderating function of family background. It is initially necessary to define the categories of similar CP events. This process followed an inductive approach and was conducted through factor analysis on the basis of the respondents’ answers to the questionnaire, resulting in a series of sub-groups of correlated receptive events. Once the categories were defined, aggregated measures were built for each category of cultural event. Several quantitative methods can be used for this purpose [18], and Latent Profile Analysis (LPA) was used in this instance, similarly to the methodology proposed by Mak, Coulter and Fancourt [49]. As a result, the respondents were clustered by participation frequency in all events, making it possible to identify homogeneous groups in the population according to their cultural participation. Thereby, the respondents belonging to the groups with relatively higher participation frequency were assumed to be participants, and those with lower frequency were assumed to be non-participants.

Propensity Score Matching (PSM) is then chosen to assess the relationship between CP and life satisfaction. This model has been considered marginally in extant literature on cultural studies, but is gaining attention in the academic world because of its quasi-experimental approach that gives a closer estimate of a causal relationship than traditional models [63]. This methodology addresses the existing confounding biases, thus isolating the relationship between cultural participation and life satisfaction from the influence of the other covariates. For instance, while we may observe with traditional models a positive correlation between cultural engagement and life satisfaction, it could be in part explained by a third variable, such as people’s education. We can’t exclude, with traditional models, that the positive correlation that we observe is explained by the fact that people who have higher degrees are more likely to engage, and also to be more satisfied with their lives. PSM addresses this potential threat by removing any influence on the association from observed confounding variables included in the analysis. Specifically, PSM works by identifying two groups in the sample of respondents based on a pre-defined treatment (cultural participation in this case). One group consists of the respondents subjected to the treatment, and the other is the control group. Random assignment can be simulated through what is referred to as a matching process, whereby each person subjected to the treatment is paired with a person in the control group whose probability of receiving the treatment, namely “propensity score" (PS), is virtually the same. The PS is thus defined through a set of confounding variables that should be as large as possible to ensure a reliable fit. In our case, PSM is used to compare the difference in life satisfaction between two people who are virtually identical apart from choosing to take part or not to take part in cultural events, simulating an experiment where cultural participation (i.e., the “treatment”) is assigned randomly. In this process, the sample is balanced through what is known as the caliper width, which is the range within which the scores of two matched observations must fall. As a final result, it is necessary to test that there is no statistical difference between the treated and control samples for all the confounding variables (i.e., the p-values must be as high as possible). As described in the previous section, this study included a different set of confounders for the matching process: family background, income range, age, gender, level of education, type of job/employment, geographical area of residence, population of the town/city of residence, religious faith and household size.

For the matching process, three different methods were compared to provide more robustness to the analysis. At first, nearest neighbour matching (NNM), which is the most commonly used and recursively assigns each treated unit to the control with the closest PS [64]. Instead, optimal matching (OM) method minimises the total distances among all matching unit [65]. The third method is full matching (FM) forms sub-classes in the sample with at least one treated and one control unit, minimising the within-class differences [66]. In contrast to the others that perform a 1:1 match, this latest method provides a weight for each unit in the dataset. Those three method were then compared along three main metrics to select the most effective one, following literature on the subject [63,67]. Firstly, as mentioned before, the ability to generate two identical groups in terms of the covariates considered. Then, we expect that after the matching the correlation between the treatment (i.e., cultural participation) and the confounding variables is minimised. Finally, we aimed at maximising the effective sample size (ESS), namely the final dimension of the dataset with the final set of matched units (or, in the case of FM, the effective dimension of the weighted dataset).

Once the pairing process was conducted and validated, the model compared the difference in outcomes between participants and non-participants, measuring the Average Treatment Effect (ATT) on the treated sample. In this step, simple OLS was employed. The matching process and the subsequent ATT measurement were run for each category of CP identified in the previous step, identifying the average effect of each category on the whole population analysed.

In the second step, the study distinguishes the effects of cultural participation given people’s family background. In order to test this moderation exerted by family background, it is necessary to capture in a more comprehensive model the mixed effect of family background and categories of cultural participation on the respondents’ life satisfaction. To carry out this exercise, the simple PSM model used in the first step was extended, in line with recent studies looking into the possibility of introducing two simultaneous “treatments” [63]. While inequalities of opportunity are traditionally measured through ex-ante or ex-post approaches [25], this novel methodological approach leverages on PSM’s ability to limit confounding biases. As a result, through PSM the study describes at the same time the existing inequalities of opportunities – comparing the life satisfaction of different family backgrounds – and the role of cultural participation in offsetting (or exacerbating) those existing imbalances. In other words, this model is able to capture simultaneously the link between cultural participation and life satisfaction for people coming from advantaged backgrounds, and the same link for people from disadvantaged backgrounds.

Technically, in this model, people’s background was considered as an additional factor that could affect their outcomes, and is expressed as a dichotomous variable (“0” for disadvantaged background, “1” for advantaged background). The overall sample was thus divided into four groups, given by the four possible combinations of background and CP (namely, people from disadvantaged backgrounds who do not engage in cultural events, people from disadvantaged backgrounds who do engage in cultural events, people from advantaged backgrounds who do not engage in cultural events, people from advantaged backgrounds who do engage in cultural events). Just as the methodology applied in the original PSM models [68], the algorithm then measures the propensity scores for each group (i.e., the probability of belonging to each of the four groups) by means of a multinomial logit model. The similarity index for each respondent, was thus based on the probability of belonging to each of the four groups, given his/her socio-demographic and economic characteristics. This is defined as follows for each respondent *i*:


$$P_{ik}=\frac{e^{x_{i}^{\prime }\beta_{k}}}{1+\sum_{l=1}^{K}e^{x_{i}^{\prime }\beta_{l}}},$$ (1)

where $\beta_{k}$ is the vector of regression coefficients for each of the *x*′*_i_* covariates along the four *k* groups.

The matching process was then conducted using a model aligned to the one used in the first step of the analysis. In this case, we measured the distance between observations through logarithms of ratios (logratios), in that these provided the best fit in comparison to the Euclidean and Manhattan distances. The maximum distance between the matched observations was thus set in terms of maximum logratio distance, and follows the same logic as the caliper width. The results reached on considering the combined effect of CP and background address the second research question.

## Results

The results are arranged in the following way. The first subsection identifies the various categories of cultural participation events. Then, we set out the average association between cultural participation categories and life satisfaction for the whole population and, lastly, the moderating role of family background on life satisfaction, thus distinguishing between the effect to advantaged and advantaged backgrounds, is examined.

### Cultural participation categories

The objective is to establish who to place in the group of participants and who in the group of non-participants. LPA modelling is used for this purpose to determine how frequently each person participated in cultural events, as detailed in Materials and Methods section. Because of the variety of possibilities that fall within the concept of receptive cultural participation, it is necessary to find a comprehensive and synthetic measure that could ascertain whether a person participates in cultural events or not.

**Fig 2:**
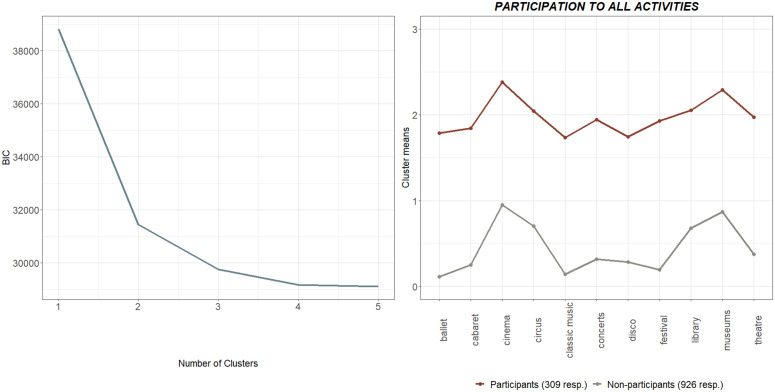
Clusters of overall cultural participation. BIC by number of clusters and cluster means created according to CP frequency.

Before identifying the various categories of similar events, a metric combining all 11 events is set up in the study, with the purpose of providing an aggregated measure more similar to those used in previous studies [38,49], especially given the high reliability of the overall construct (Cronbach α = 0.943). Considering the AIC, BIC and Entropy metrics (BIC metric dependent on the number of clusters is shown on the left-hand side of Fig 2), two clusters are deemed as suitable to grasp most of the variability. As a result, looking at the cluster means on the right-hand side of Fig 2, there is one cluster where there are low participation rates in all events and another where the respondents are instead highly engaged in cultural events. The cluster of non-participants, which is the largest with 926 respondents, has low mean values of participation in all the events considered, with a slightly higher attendance frequency for cinema, circus and museums, indicating occasional visits. As a result, CP is now expressed in a binary form for each individual, and is equal to “1” if that person belongs to the cluster of participants and to “0” otherwise.

**Fig 3:**
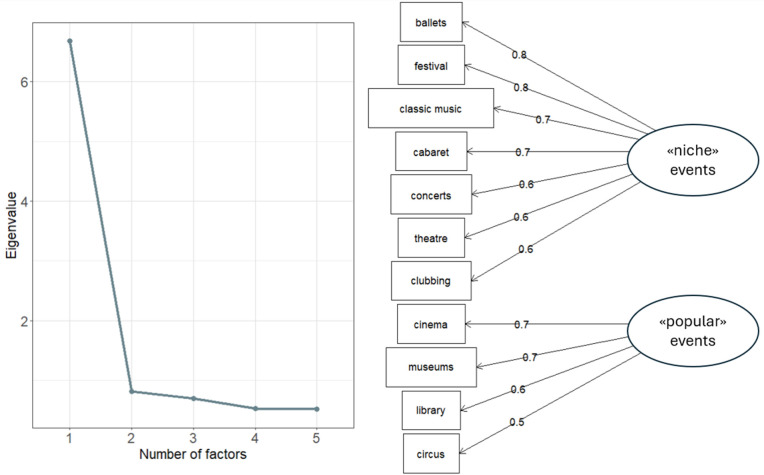
Identification of cultural participation categories. FA eigenvalues and activities grouped with two factors.

Factor analysis is then used to assess whether the 11 events in the study can be classified into homogeneous sub-groups. Looking at the resulting eigenvalues, this method clearly identifies two separate categories, as shown in Fig 3. The first category includes cinema, museums, library visits and the circus. Referring to the outcomes of the LPA carried out previously (Fig 2), these four are apparently the most popular events, attended occasionally even by the non-participants (at least once a year). The remaining events make up the second category, they are more selective and enjoyed by a certain audience. Many of them, and specifically the ballet, theatre and classical music in particular, are seen as highbrow [8,69]. At the same time, this group also includes economically accessible events connected to urban/underground culture, such as stand-up comedy festivals. All the events in this group share a common feature, in the sense that there is a definite intention on the part of the participants to take part in the cultural experience, and participation generally appears to be more socially stratified [33]. For this reason, we have called the first category the “popular” CP group, and the other category is the “niche” CP group, to indicate that these events are not exclusively part of traditional highbrow culture, but are rather experiences for a more discerning and stratified audience.

**Fig 4:**
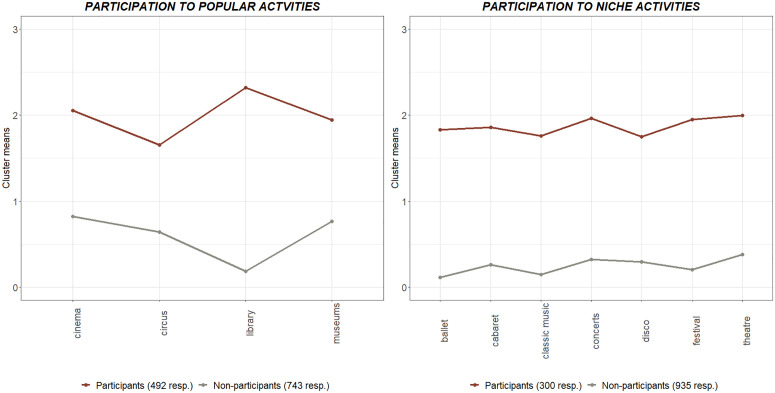
Cluster means for popular and niche CP events.

The two categories of cultural events are again analysed through LPA, following the same procedure used to analyse all 11 events together. Two other dichotomous CP measures are thus defined, representing “popular” (Cronbach α = 0.800) and “niche” (Cronbach α = 0.929) cultural participation, taking into account only frequency of participation to the events in either category. The cluster means for “popular” and “niche” cultural participation are shown in Fig 4. Looking at the size of the two clusters, as to be expected, more people apparently take part in popular events than in niche events.

### Relationship between CP and life satisfaction

The aim of this part of the analysis is to estimate the overall effect of cultural participation on life satisfaction, achieved through Propensity Score Matching (PSM).

The first step in a PSM exercise consists of running a matching process, where each “treated” observation (in this case, each participant) is paired with a “control” observation (i.e., a non-participant) that is virtually identical when all possible confounding variables are taken into account. This process is controlled by setting a maximum caliper distance (expressing the difference between the two matched units) equal to 0.2, a standard setup in extant literature [70]. For each of the three measures of cultural participation (overall, popular and niche), three different matching models are compared (NNM, OM and FM). While all models successfully generated identical treated and control groups (p-value greater than 5% for all confounders), some differences emerged while evaluating the correlation with (continuous) confounders and the effective sample size (ESS). In fact, OM appears to perform worse than the others in terms of confounders correlation (which should be as close as 0 as possible), whereas full matching provides a lower ESS. The comparison between the three machining methods is available in Tables 2, **S3 Table** and **S4 Table** (the latter two in the Supporting information section).

**Table 2:** Matching models comparison for overall CP.

Confounder corr.	original data	NNM	OM	FM
*Age*	−0.210	−0.012	−0.211	−0.014
*Education level*	0.076	0.012	0.075	0.000
*Income range*	0.038	−0.002	0.037	0.009
*Household size*	0.072	−0.026	0.073	0.009
*Family Background*	0.191	0.028	0.192	0.003
*City size*	0.056	0.030	0.056	−0.018
ESS		NNM	OM	FM
		502	536	375

The two groups are thus matched using the NNM without-replacement algorithm. Three distinct matching processes are run, one with the set of all 11 CP events (Model 1) to give the general baseline, one with the popular CP events (Model 2) and one with the niche CP events (Model 3). The matching process excluded the missing observations for income range. The study thus includes the 10 confounding variables listed in the Database section. At the end of the matching process, 516 participants are included successfully in Model 1, 760 in Model 2 and 502 in Model 3.

After completing the matching process, it is possible to assess the overall Average Treatment Effect on the Treated (ATT) of cultural participation on life satisfaction. Ordinary Least Squares regression (OLS) is applied three times, to analyse the impact of all CP events, then of popular CP events and lastly of niche CP events. The results are given in Table 3. In all three models, the relationship between cultural participation and life satisfaction (the first three rows in Table 3) has a positive and significant coefficient. Comparing the coefficient of the CP variables, it appears that going to niche events has the strongest association with life satisfaction. Looking at the results obtained from the models, the other significant variables are income, household size and, to some degree, family background and faith. Moreover, looking at the three models, R-squared is highest in the niche participation model, indicating that going to such events plays a major part in explaining higher life satisfaction.

**Table 3:** Overall relationship between CP categories and life satisfaction.

	*Life Satisfaction (y)*		
	(1)	(2)	(3)
CP (All events)	2.377^***^		
	(0.497)		
CP (Popular)		1.732^***^	
		(0.415)	
CP (Niche)			2.046^***^
			(0.514)
Income	0.671^***^	0.785^***^	0.470^**^
	(0.173)	(0.140)	(0.175))
Age	0.006	0.009	0.035
	(0.024)	(0.019)	(0.024)
Gender_F	−0.146	−0.217	0.304
	(0.520)	(0.427)	(0.536)
Region_NorthEast	−0.990	−0.496	−1.175
	(0.760)	(0.608)	(0.817)
Region_Center	−1.005	−0.501	−0.453
	(0.809)	(0.636)	(0.849)
Region_South	−0.814	0.044	−0.289
	(0.736)	(0.614)	(0.769)
Region_Islands	0.493	1.107	0.365
	(0.885)	(0.743)	(0.917)
Education level	−0.474	−0.294	−0.007
	(0.258)	(0.212)	(0.269)
F. Background_2	0.621	0.550	2.570^*^
	(1.219)	(0.834)	(1.208)
F. Background_3	1.660	1.228	3.538^**^
	(1.179)	(0.781)	(1.184)
F. Background_4	0.486	0.619^*^	0.253
	(0.327)	(0.256)	(0.341)
City size	−0.006	−0.015	−0.110
	(0.174)	(0.139)	(0.179)
Household size	0.726^***^	0.383^*^	0.775^***^
	(0.203)	(0.179)	(0.218)
Faith_atheist	−0.704	−0.891	−0.919
	(0.619)	(0.516)	(0.627)
Faith_agnostic	−1.767^*^	−1.206	−2.854^**^
	(0.806)	(0.664)	(0.893)
Faith_no answer	0.571	−1.279	−0.156
	(1.303)	(1.093)	(1.464)
Occupation_student	−2.209	−1.162	−1.789
	(2.530)	(1.526)	(2.452)
Occupation_unemployed	−0.685	−1.040	−1.148
	(2.505)	(1.478)	(2.441)
Occupation_worker	−1.686	−1.221	−1.495
	(2.294)	(1.151)	(2.187)
Observations	516	760	502
R^2^	12.6%	10.0%	12.6%

Note: ^*^p<0.05; ^**^p<0.01; ^***^p<0.001

### The moderating role of family background

This section explores the role of family background in moderating the ability to achieve higher life satisfaction through cultural participation is then examined in the second part. The three previous models have been extended to take in the combined association of cultural participation and family background with life satisfaction.

For the purposes of this investigation, traditional PSM has been extended by the authors to take in the simultaneous effect of two “treatment” variables, cultural participation, CP, and family background, FB. FB is now expressed as a binary variable, where “0” indicates a lower level of parental education (lower secondary school/middle school or below for both parents), and “1” indicates a more advantaged background (upper secondary school/high school or above). The three CP measures built in the previous section are retained. According to the four possible combinations of the two binary variables, the new PSM model matches respondents in quadruples through NNM, creating four statistically identical groups (p-value greater than 5% for each confounder), each one with a different combination of CP and FB. Lastly, similarly to the procedure run on the traditional PSM model in the previous section, the differences in life satisfaction for the different groups are assessed through an OLS regression, which includes all the confounders considered in the study. This methodological approach allows to limit the existing confounding bias that could affect the coefficients of both cultural participation and family background simultaneously.

Four groups of respondents are created taking the CP variable that includes all events, distributed as follows: 300 obs. with FB = 0 ∧ CP = 0, 72 obs. with FB = 0 ∧ CP = 1, 439 obs. with FB = 1 ∧ CP = 0, and 196 obs. with FB = 1 ∧ CP = 1. The matching process came up with 60 quadruples of virtually identical respondents (p-value greater than 5% for all confounders). The results of the OLS are shown in Table 4, indicating the effect of the different combinations of FB and CP on life satisfaction. On comparing the different groups, it appears that life satisfaction is particularly low in the group of disadvantaged people who do not take part in cultural events and, additionally, it is significantly lower than in the other groups. In other words, the decision to attend cultural events is related to an increase of 3.923 points in the life satisfaction scale. Looking only at advantaged backgrounds, no significant difference is instead found between participants and non-participants.

**Table 4:** Association of CP (all activities) and FB with life satisfaction.

OVERALL CULTURAL PARTICIPATION				
	*Life Satisfaction between Groups:*			
	Disadvantaged FB.		Advantaged FB.	
	*CP = 0*	*CP = 1*	*CP = 0*	*CP = 1*
*baseline*	(-)	(CP)	(FB)	(CP, FB)
(-)		3.923^***^	3.811^***^	3.201^**^
		(1.118)	(1.125)	(1.185)
(CP)			−0.112	−0.722
			(1.125)	(1.168)
(FB)				−0.609
				(1.150)
Observations:		240		
R^2^		20.04%		

Note: ^*^p<0.05; ^**^p<0.01; ^***^p<0.001

The same four-group model as before is run using the popular CP group and then again using the niche CP group. For popular CP events, the numbers are 254 obs. with FB = 0 ∧ CP = 0, 118 obs. with FB = 0 ∧ CP = 1, 352 obs. with FB = 1 ∧ CP = 0, and 283 obs. with FB = 1 ∧ CP = 1. For niche CP events, they are 301 obs. with FB = 0 ∧ CP = 0, 71 obs. with FB = 0 ∧ CP = 1, 447 obs. with FB = 1 ∧ CP = 0, and 188 obs. with FB = 1 ∧ CP = 1. At the end of the matching process, 112 and 63 quadruples are selected, for the popular and niche group respectively. The two regression models are then conducted, and the results are given in Table 5 and Table 6. Investigating the moderating role of family background, the results confirm that it plays a significant role in affecting the effect of cultural participation on life satisfaction. People from disadvantaged backgrounds appear to benefit significantly both by niche CP (+2.977) and, to a smaller extent, by popular CP (+1.932). People from an advantaged background who take part in niche cultural events have no significant difference in levels of life satisfaction, while attending only popular CP events seems to generate some significant effect (+1.791).

**Table 5:** Association of CP (Niche) and FB with life satisfaction.

NICHE CULTURAL PARTICIPATION				
	*Life Satisfaction between Groups:*			
	Disadvantaged FB.		Advantaged FB.	
	*CP = 0*	*CP = 1*	*CP = 0*	*CP = 1*
*baseline*	(-)	(CP)	(FB)	(CP, FB)
(-)		2.977^**^	3.376^***^	3.399^***^
		(0.978)	(1.001)	(1.023)
(CP)			0.399	0.423
			(0.972)	(1.005)
(FB)				0.023
				(1.006)
Observations:		252		
R^2^		17.0%		

Note: ^*^p<0.05; ^**^p<0.01; ^***^p<0.001

**Table 6:** Association of CP (Popular) and FB with life satisfaction.

POPULAR CULTURAL PARTICIPATION				
	*Life Satisfaction between Groups:*			
	Disadvantaged FB.		Advantaged FB.	
	*CP = 0*	*CP = 1*	*CP = 0*	*CP = 1*
*baseline*	(-)	(CP)	(FB)	(CP, FB)
(-)		1.932^*^	0.765	2.556^**^
		(0.791)	(0.793)	(0.822)
(CP)			−1.167	0.624
			(0.792)	(0.809)
(FB)				1.791^*^
				(0.811)
Observations:		448		
R^2^		10.81%		

Note: ^*^p<0.05; ^**^p<0.01; ^***^p<0.001

## Discussion

This study is concerned with investigating the relationship between cultural participation and life satisfaction, assessing how it is moderated by family background. With reference to cultural participation, recent studies are starting to investigate this impact variable on life satisfaction, showing early significant results, but also contradictory findings in the intensity of the links, suggesting further investigation is needed to disclose the main influencing factors [3,10,11,20]. Looking at the various factors that have an influence on cultural participation, one authoritative voice comes from the Bourdieusian stream of thought and, while concentrating on income, suggests that family background is a key driver for access [9,32]. Conceptually rooted in [27] view on inequalities of opportunity and highlighting the influence of exogenous factors on a person’s individual life satisfaction, this study adds to previous research at two levels.

The first contribution relates to a clearer understanding of how diversity among events influences their effect on life satisfaction, primarily by identifying possible differences between receptive CP events. In the existing literature, the question of the heterogeneity of cultural experiences, and of whether some events are more likely to be more significantly related than others to life satisfaction is still under debate [10–12]. Despite the diversity of cultural expressions, the events considered in this study observe coherent and aligned patterns, making it possible to build meaningful latent constructs that can distinguish participants from non-participants in the sample. Two sub-categories of receptive cultural experiences were identified through a novel inductive approach. One group consists of “popular” events, such as visits to museums, libraries, the cinema, the circus and open-air cultural events, all of which are well-attended by a large audience, and occasionally attract even the least engaged population. The other group consists of “niche” events, a variegated selection that includes ballet, music festivals, classical music, rock and pop concerts, cabaret, clubbing and theatre shows, which offer a more selective and stratified experience [33].

Having put in place these definitions, we investigated the link between cultural participation and life satisfaction via PSM, finding a positive and significant relationship, in line with several studies [10,11,16]. However, while the positive association of cultural participation emerges when all cultural categories are taken into consideration in the study, niche events appear to generate the strongest effect on the respondents’ life satisfaction. These findings are in line with studies that have previously addressed this topic, but also provide more detailed insights into the diversity of cultural events, which are instead often considered individually [11,20] or in an aggregated construct [21,38,49]. In general, the higher effect of niche CP compared to popular CP highlighted in this study can be linked to [10,16,47], who observed that arts initiatives have a stronger impact on life satisfaction than museums and libraries. However, separating cultural events into two categories, as proposed in this study, helps with the generalisability of the results.

**Fig 5:**
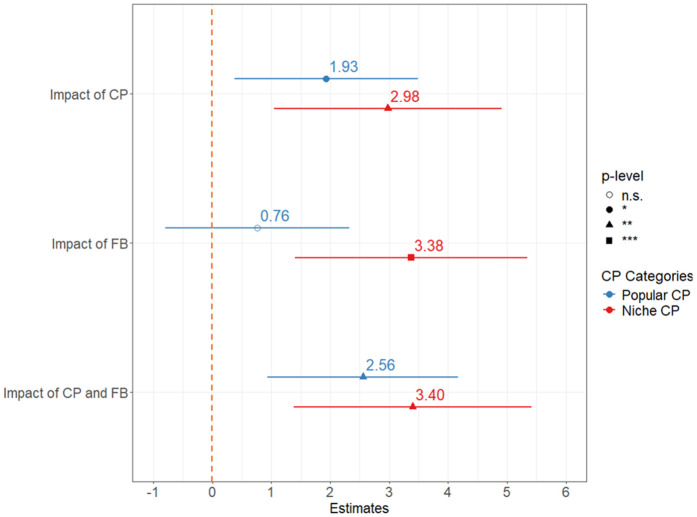
The combined influence of family background and cultural participation on life satisfaction.

The second main finding relates to the moderating role of family background, a contribution shown to affect a person’s social capital and preferences [1,22,71]. It was confirmed that this variable is a relevant factor that affects not only income levels, as theorised originally, but also life satisfaction. The moderating effect of family background on life satisfaction is given in Fig 5, where the reference is life satisfaction in non-participants from lower family backgrounds (red dashed line). In general, the impact of CP appears to be significant and strong especially for people from a disadvantaged background. While their life satisfaction is considerably lower than that of the rest of the population when they do not take part in cultural events, this imbalance disappears when they instead join in culturally. Interestingly, people who attend cultural events enjoy the same level of life satisfaction, regardless of their background. There is no cumulative effect of an advantaged background and cultural participation on life satisfaction, as only one of the two can set the process in motion, thus aligning the population’s life satisfaction as a whole. These findings highlight the fact that culture provides an opportunity to address unfair inequalities in life satisfaction that depend on a person’s background. Although this consideration seems generally true regardless of the category of cultural event, the effect of niche CP seems to be stronger in disadvantaged backgrounds than popular CP (+2.977 vs + 1.932).

Focusing instead on people from an advantaged family background, studies in the footsteps of Bourdieu’s seminal work have often outlined that, when upper classes indulge in cultural participation, they gain in terms of social distinction [22,71]. While this theory has been tested for the participants’ level of income, the results presented in this paper on life satisfaction are not as obvious. While we can flag up some distinctions for people from lower backgrounds who do not take part in cultural events, we do not find a net difference in life satisfaction between culturally active participants in this group and everyone in the other, advantaged, group. The only marginally significant effect for people from an advantaged background seems to be given by them participating in popular events, these being the most common within the population and the least stratified. The weak benefit linked to an advantaged background suggests that there are potentially some differences between the purely materialistic perspective of social distinction [8], measured through income, and the quality-of-life perspective, measured through life satisfaction. Moreover, the fact that the association between cultural participation and life satisfaction is only significant for a part of the population (i.e., mainly those from a disadvantaged background) could explain why a strong association with life satisfaction was not always observed in previous studies and for some activities [10,16,21].

## Conclusion

For some years now, the existence of inequalities of opportunity has been put at the centre of the academic and practitioner debate, and there is the need to disclose democratising factors actionable by public policy. This study contributes to advancing research in this area by focusing on the relationship between cultural participation and people’s life satisfaction, considering their acquired family background. Starting from the results discussed in the latest research on the subject, this article highlights how a more comprehensive and precise investigation into relevant factors such as family background and the breakdown of cultural events into separate categories, can unveil additional neglected elements. Two categories of events were identified through a rigorous inductive approach, niche and popular events. The effect of taking part in cultural events on life satisfaction was found to be higher for niche events, which consist of more selective and stratified experiences, such as going to the theatre. Moreover, when exploring how to address the imbalances in life satisfaction within the population and promote equality of opportunity, niche cultural events were found to be the most relevant for people from more disadvantaged backgrounds. Taking part in cultural events can offset structural inequalities in life satisfaction between these people and those from advantaged family backgrounds.

Along with these encouraging results, some limitations and opportunities for further research can be identified. Firstly, while PSM is able to isolate the relationship between cultural participation and life satisfaction variables, it does not fully address endogeneity issues. Thus, causality can’t be claimed, and future investigations could address this existing gap. We also highlight that the results are bound to the Italian context, and this could have influenced, for instance, the classification of cultural events into “popular” and “niche” categories. Further studies could replicate the same analysis in different countries to verify whether the most relevant events for disadvantaged people are the same. Moreover, the most disadvantaged population was only partially considered in our study, owing to the difficulty of reaching these people through internet-based surveys (CAWI), thereby leading to an under-representation in responses. Future research could replicate this investigation by specifically addressing the most vulnerable groups with ad-hoc investigations to extrapolate stronger evidence on the nature and significance of the relationship investigated for these minorities.

In general, the study’s findings present some practical implications by supporting the design of data-driven and evidence-based policies. Showing how participating in cultural events can be a significant opportunity to affect individuals’ life satisfaction, the study may suggest that policies addressing the promotion of cultural events should not overlook their potential in advancing socio-economic development. Policy design would thus need to leverage on the variables that encourage cultural participation and thus could significantly reduce differences in well-being among social classes. Accessibility to cultural events is a key element. In areas with high social inequalities and lower household conditions, when the authorities design cultural policies, they could look at how to promote accessible cultural events that target the most disadvantaged households, favouring the improvement of their life satisfaction.

## Supporting information

S1 Table Descriptive statistics and PSM balancing. (PDF)
