# Supplementary material for: Cultural participation and life satisfaction: an investigation across different family backgrounds
Source: PLoS One. 2026 Apr 10;21(4):e0346887. doi: 10.1371/journal.pone.0346887 (PMC13068270; doi:10.1371/journal.pone.0346887)
Supplement: S1 Table — (PDF) [file pone.0346887.s001.pdf]

Cultural participation and life satisfaction: an investigation across different family backgrounds

Romain Lerouge[1,*], Michela Arnaboldi[1]

March 27, 2026

1 Management Engineering, Politecnico di Milano, Milano, MI, Italy
***** Corresponding author
E-mail: romain.lerouge@polimi.it

Supporting information

S1 Table.Descriptive statistics and PSM balancing

Variables	N	%
Museum visits	1 235	
Never		35.2
Once in the past year		20.1
A few times in the past year		31.2
A few times a month		13.5
Classical concerts and opera	1 235	
Never		69.5
Once in the past year		12.4
A few times in the past year		12.5
A few times a month		5.7
Theatre shows	1 235	
Never		54.8
Once in the past year		18.4
A few times in the past year		20.9
A few times a month		5.9
Ballet and traditional dance performances	1 235	
Never		68.7
Once in the past year		13.6
A few times in the past year		12.9
A few times a month		4.8
Music and dance festivals	1 235	
Never		62.3
Once in the past year		17.0
A few times in the past year		15.5
A few times a month		5.2
Cabaret and stand-up comedy	1 235	
Never		62.2
Once in the past year		15.6
A few times in the past year		16.8
A few times a month		5.3
Cinema	1 235	
Never		36.9
Once in the past year		14.3
A few times in the past year		29.6
A few times a month		19.2
Pop and rock concerts	1 235	
Never		49.6
Once in the past year		13.8
A few times in the past year		20.8
A few times a month		15.8

Variables	N	%
Library and archives	1 235	
Never		59.5
Once in the past year		14.9
A few times in the past year		18.9
A few times a month		6.6
Disco	1 235	
Never		67.0
Once in the past year		9.8
A few times in the past year		14.0
A few times a month		9.1
Circus and open-air cultural events	1 235	
Never		42.6
Once in the past year		20.1
A few times in the past year		26.2
A few times a month		10.3
Occupation	1 235	
Entrepreneur/business owner		3.0
Self-employed professional		4.9
Director, upper management		2.1
Middle management		2.7
White-collar worker (permanent contract)		30.8
White-collar worker (other contract)		4.1
Merchant/trader of a commercial operation		1.6
Manual worker/artisan		5.1
University lecturer		0.4
Teacher (primary and secondary schools)		3.2
Officer in the armed forces or police		0.6
Other Members of armed forces or police		0.7
General practitioner		0.7
Medical registrar		0.2
Farm owner/manager		0.2
Farm worker/labourer		0.2
Home maker (no outside occupation)		8.7
Pensioner		5.7
Unemployed or job seeker		7.3
Student		8.0
Other		9.7

PSM diagnostics Matching models comparison for popular CP

Confounder corr.	original data	NNM	OM	FM
Age	-0.128	-0.014	-0.024	0.010
Education level	0.097	0.014	0.017	-0.029
Income range	0.059	-0.002	-0.009	-0.031
Household size	0.101	0.033	0.053	0.023
Family Background	0.146	0.017	0.032	0.017
City size	0.029	0.015	0.024	0.025
ESS		NNM	OM	FM
		762	802	575

PSM diagnostics Matching models comparison for niche CP

Confounder corr.	original data	NNM	OM	FM
Age	-0.210	-0.031	-0.029	-0.009
Education level	0.064	-0.014	-0.021	-0.024
Income range	0.037	-0.007	-0.009	-0.028
Household size	0.056	0.021	0.005	0.021
Family Background	0.183	-0.021	-0.018	0.009
City size	0.044	-0.033	-0.032	-0.023
ESS		NNM	OM	FM
		498	518	427
